# Does report modality modulate psychophysical sensitivity? The jury remains out

**DOI:** 10.3389/fpsyg.2023.1226588

**Published:** 2023-10-19

**Authors:** Oliver J. Hulme, Barrie Roulston, Morten Overgaard

**Affiliations:** ^1^Danish Research Centre for Magnetic Resonance, Copenhagen University Hospital Amager and Hvidovre, Copenhagen, Denmark; ^2^London Mathematical Laboratory, London, United Kingdom; ^3^Department of Psychology, University of Copenhagen, Copenhagen, Denmark; ^4^Independent Researcher, London, United Kingdom; ^5^CNRU, Center for Functionally Integrative Neuroscience, Aarhus University, Aarhus, Denmark

**Keywords:** report, conscious, modality, action, methodology

## Abstract

Scientific studies of perception use motoric reports as the principal means of communicating subjective experience. In such experiments, a widely held and implicit assumption is that the motor action conveys but does not tamper with perceptual experience. We tested nine observers on a luminance detection task in a cross-over repeated measures design. In separate conditions, observers reported their detection via movements of either their hands or eyes. We found only anecdotal evidence for any modality-dependent effect on psychophysical sensitivity. We also reanalyzed an existing dataset from which deployed a similar detection paradigm involving hand and eye reports. In the four paradigm variants tested, we again only found anecdotal evidence for the effect of report modality on psychophysical sensitivity. Both studies reported here provide only anecdotal evidence; thus, whether we can replicate report-dependent perceptual effects still needs to be resolved. We argue why this remains an important question for consciousness research and why it deserves more rigorous and high-powered replication attempts.

## Introduction

**Figure 1:**
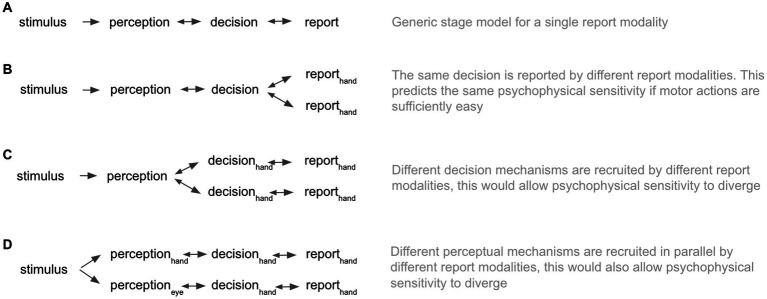
Stage models of conscious perception. **(A)** Illustrates a generic stage model for a simple detection task with a single report modality. **(B)** Illustrates a stage model with two report modalities, which report on the content of a unified decision mechanism. **(C)** Illustrates a stage model in which separate decision processes and downstream reports form in parallel. **(D)** Illustrates a stage model in which the perceptual, decision and report processes are parallel.

In the cognitive and perceptual sciences, it is common to instruct observers to disclose motoric reports as an index of their subjective experience (e.g., Friston et al., 1995; Leopold and Logothetis, 1996; Genovese et al., 2002; Lamme, 2003; Hulme et al., 2009; Overgaard and Grünbaum, 2011; Overgaard and Sandberg, 2012; Tsuchiya et al., 2015; Andersen et al., 2016; Skewes et al., 2021; Andersen et al., 2022). A widely held and implicit assumption is that this sensorimotor arc begins with stimulus input, unfolds as a perception, and results in a decision that culminates in a motoric report (Figure 1A). Here, the report is the final stage conveying the semantic information pre-specified by the task. In such stage models of cognition and consciousness (Overgaard and Mogensen, 2017), one can compare results from experiments that have used different report modalities since the means of the report should not influence the earlier perceptual stages (Figure 1B). Under this model class, the assumption of report-modality invariance follows intuitively from the fact that reports are “temporally and logically posterior to the perceptions they describe” (Marcel, 1993).^1^ In conflict with this view, Marcel (1993) reported that observers’ psychophysical sensitivity in a simple speeded detection task varied according to the report modality. Observers performed a visual detection task, simultaneously reporting via three different modalities. Eye blinks were the most sensitive (mean d’ 0.91), followed by finger-presses (mean d’ 0.85), and then verbal reports (mean d’ 0.18).^2^ In a second condition where subjects were instructed to guess, Marcel reports the same ordering in the sensitivity of report modalities (eye-blink mean d’ 2.07; button-press mean d’ 1.76; verbal mean d’ 1.52). These results would be predicted under either a model in which different report modalities recruit different decision-making pathways, which could be subject to different noise levels (Figure 1C) or a model in which perception itself is somehow contingent on the report modality (Figure 1D). Despite these original findings being theoretically intriguing, it is important to note that the author reported no inferential statistical tests in the original paper. Thus, the interpretation rests entirely on differences between descriptive statistics. Based on the original report, we could not recover the information necessary to calculate inferential statistics.

Report-dependent perceptual phenomena (RDPP) such as that claimed in Marcel (1993) echo earlier clinical studies showing that patients may have access to different levels of sensory information depending on which response modality they use. For instance, patients with visual extinction after brain damage to the right hemisphere were significantly worse at detecting contralesional stimulus when using a keypress report than when using a verbal report (Bisiach et al., 1989). Joanette et al. (1986) report how the subjective report of visual stimuli in hemispatial neglect patients depends on the hand used to report. All patients in their study reported more stimuli when reporting with the left hand (controlled by the contralesional hemisphere) than the right. In contrast, psychophysical sensitivity was the same for both hands. Related findings have been reported in a patient with a focal anterior cingulate cortex lesion whose performance on Stroop and divided attention tasks depended on the response modality used (Swick and Turken, 1999). The patient exhibited impairment in manual responses but not vocal responses under the same task requirements. Beyond neuropsychology, Gomi et al. (2013) showed that visual motion coding responds differently according to particular downstream motor outputs, with hand reports being less sensitive to the central occlusion of visual motion than eye reports. In all these cases, motor outputs modulate visual thresholds. Further to this neurobiological plausibility, a large body of theoretical work on action-perception loops (Gibson, 1979; Clark, 2015) conjectures a dynamic and bidirectional relationship between perception and action as fundamental to cognition. Such models would naturally predict a strong dependence between the action networks that are deployed to report a percept and the perceptual networks responsible for the perception. Closely related to this is the phenomenon of action-specific perception, whereby the action for which a perception is needed has a qualitative effect on the perceptual content (Witt, 2011). A commonly reported example is that softball players who are hitting better see the ball as bigger (Witt and Proffitt, 2005). As mentioned above, the possibility that reporting itself may confound the neural correlates of consciousness in experiments – or even alter the experience itself – has been expressed. In previous research (Sandberg and Overgaard, 2015; Overgaard and Sandberg, 2021), we have argued that the reporting method may influence results in the sense that people will act differently when presented with a dichotomous scale, a 100-point scale or a scale with defined scale points such as PAS. However, the experiments mentioned above indicate that this is more than a methodological concern. They suggest that there might be perceptual consequences to different types of report. In recent years, no-report paradigms have appeared as a reaction against some of the methodological challenges related to reporting, i.e., paradigms where some objective behavior is measured instead of using a report (Tsuchiya et al., 2015). No-report paradigms have been recognized as a methodologically important supplement to consciousness research but have been challenged as a “stand-alone” approach. If one wishes to study subjective experience, it is unclear which objective measure to use. How can we know that correct identification or any other measure of performance is also a measure of consciousness? It seems the only knowledge we could have comes from previous experiments finding correlations between the behavior in question and introspective observation followed by a report. Thus, the behavioral measure cannot have any higher precision than the introspective observation/report, and it depends on i.

Despite several published examples of putative RDPP, it is far from established as a known phenomenon. Given the volume of experimental data on visual psychophysics, we might expect that if RDPP does exist, researchers should report it more commonly. However, as the stage model implicitly underlies most consciousness research, it is rare that more than one motoric modality is tested within the same experiment, and even rarer that researchers directly compare report modalities. Considering the potential impact of this debate on current models of perception, it is notable (publication bias notwithstanding) that the field needs to direct effort toward replicating or generalizing RDPP. Were the findings obtained by Marcel (1993) to be verified and generalized, consciousness research would face two problems: (1) accumulated knowledge must account for the fact that evidence collected using different report methods can no longer be directly compared, and (2) the assumption that that perception is prior to and independent of the report must be reconsidered.

Toward this end, we do two things in this paper. First, we present data from a repeated measures experiment designed to test for the putative existence of RDPP. We measure psychophysical sensitivity in a visual detection task, comparing a condition in which subjects report with their hands versus a second condition in which they report with their eyes via left or right saccades. Second, we reanalyze data from Overgaard and Sørensen (2004), which measured psychophysical sensitivity in four different experiments, each comparing hand reports against eye reports. We perform Bayesian statistics for both datasets to compute evidence levels for or against effects.

## Methods

### Observers

Twelve healthy observers (4 women; mean age 23, range ± 0.6 years) with normal or corrected to normal vision participated in the study after giving written consent. Data from three observers were discarded due to technical problems with eye-tracking, leaving nine observers for whom we can compare data from the two conditions. The National Hospital for Neurology and Neurosurgery Ethics Committee, London, UK, granted ethical approval for the study. All observers gave informed consent as per the declaration of Helsinki.

### Stimuli and task

**Figure 2:**
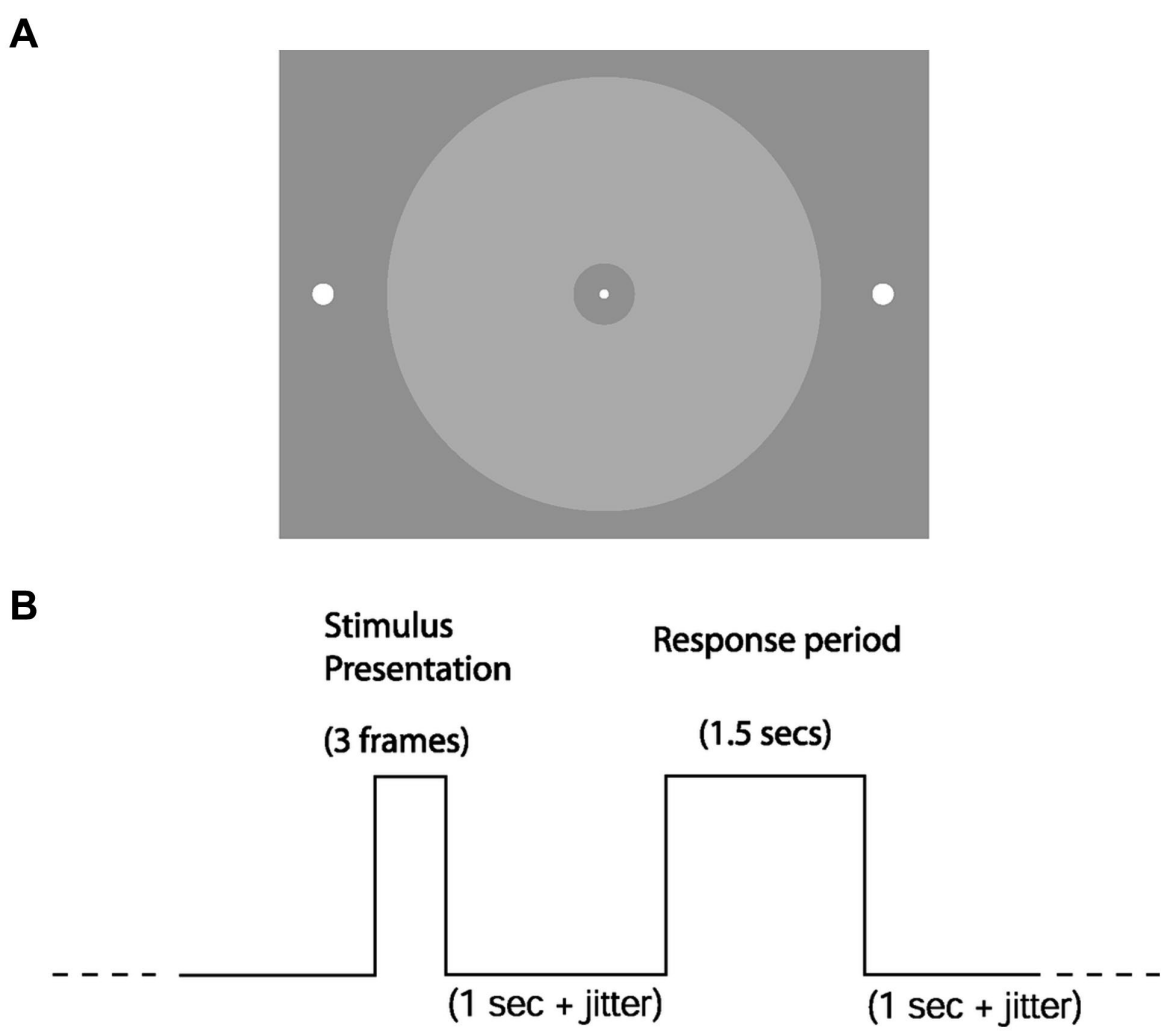
Stimuli and trial structure. **(A)** Observers were required to detect the luminance-defined disc in their parafovea. In the eye-reporting condition, they would do so by moving their eyes from the central fixation dot to either of the lateralized target dots (left to report “present,” right to report “absent”). **(B)** Shows the temporal structure of a single trial. The three frames for the stimulus presentation take 48 ms.

We made all stimuli using COGENT 2000 Graphics^3^ running in MATLAB.^4^ The stimuli were presented centrally and projected onto the screen using an LCD projector (60 Hz refresh rate). A continuous trace of horizontal eye position, vertical eye position, and pupil areas was recorded at 120 Hz using an ASL 5000 eye tracker. Observers performed two psychophysical testing sessions, one out-of-scan followed by an in-scan session, otherwise identical. We analyzed both sessions together in this manuscript. In each session, observers performed a simple detection task to obtain their psychometric performance as a function of stimulus luminance for both the saccade (eye-reporting) and keypress (hand-reporting) conditions. Each report modality was performed in separate blocks of 220 trials using the same paradigm. We permuted an equal number of (target) present and absent trials within each session. Two white dots (radius: 0.25° visual angle) were present throughout the trial on the left and right periphery. The appearance of a central white fixation point on an achromatic background indicated the start of the trial (see Figure 2 for stimulus configuration). After 800 ms plus random jitter (on the interval of 0–1 s, uniform probability distribution), either a circular achromatic ring of variable luminance (radius of ~6.2°, with an inner circular gap of ~0.3°) appeared for 48 ms (“present” trial) or did not appear at all (“absent” trial). The stimulus’s exact luminance was unknown because we could not use a photometer sufficiently near the high magnetic strength of the scanner for safety reasons. 1 s after stimulus presentation (plus jitter 0.0–0.5 s, uniform probability distribution), the fixation dot disappeared, and observers had a 1.5 s period in which to give their response (“present” or “absent”). Following that was the intertrial interval of 1 s (plus jitter on the interval of 0–0.5 s, uniform probability distribution). The keypress condition consisted of a left-hand or right-hand button press on a keypad with the index fingers of the left and right hands, respectively. For the saccade condition, observers moved their eyes to the left or right peripheral target dots before returning to the fixation dot (Figure 2A). The side representing each response (“present” or “absent”) was fixed within observers but randomly counter-balanced across observers. After fitting the psychometric function for both report modalities of each subject (separately for the in-scanner and out-of-scanner sessions), we calculated the threshold luminance value (75% detection accuracy) of the manual keypress condition.

### Data types reported

Noted there is functional neuroimaging data acquired during the in-scanner sessions. We do not report this data due to this data not being adequately analyzed when first collected and not being adequately archived to allow the data to be salvaged and reanalyzed. The primary aim of this paper rests entirely on the behavioral data acquired.

## Results

**Figure 3:**
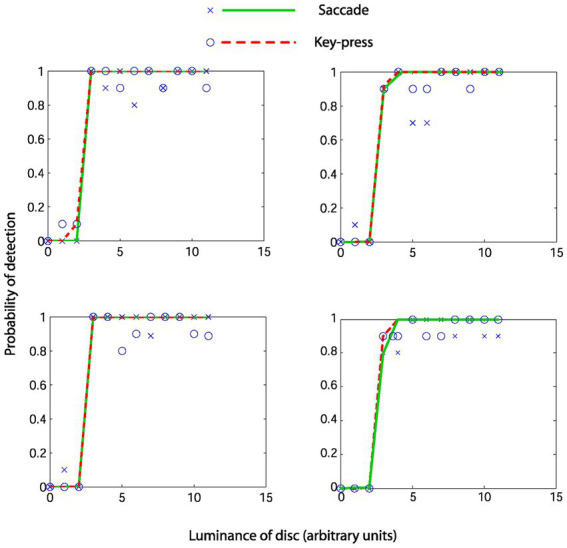
Psychometric functions from four representative observers. Crosses and circles represent real data from the saccade and keypress conditions. Solid lines represent the fitted cumulative Weibull function.

**Figure 4:**
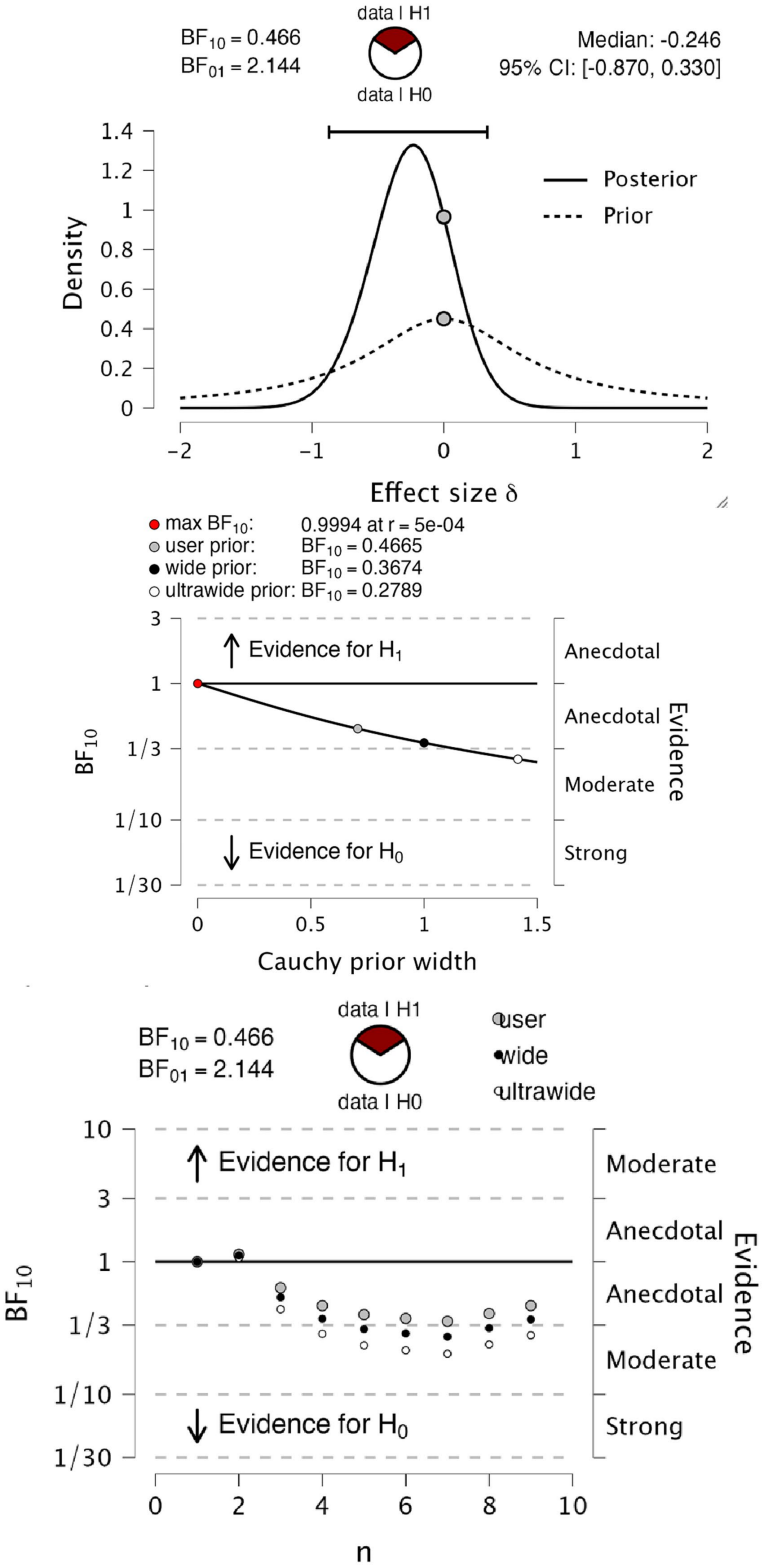
Bayesian paired *t*-test on d’. Upper panel, *t*-test information on prior and posterior values. The middle panel shows a Bayes factor robustness test for the same test: the lower panel sequential analysis of evidence levels over observer sample size.

Figure 3 shows psychophysical functions from 4 representative observers. As seen from the overlapping fits, in these four observers, the fitted psychophysical functions appear effectively the same for the two report modalities. Table 1 contains the hit rates, false alarm rates, and other signal detection measures for each participant. It can be noted that the hit rate for all participants was relatively high, approximately 90%, with a low false alarm rate below 0.01 for all participants except number 7. We do not know why this participant had a higher false alarm rate than the others. We performed a Bayesian paired *t*-test testing the null hypothesis (H0) of no difference in d’ against an alternate hypothesis (H1) of there being a difference in either direction, with default Cauchy priors with a scale parameter of 0.707 (12). We found anecdotal evidence in favor of the null hypothesis (BF10 = 0.466, BF01 = 2.144), with a median effect size of −0.246 and 95% Bayesian credibility interval (BCI95) of [−0.870, 0.330] (Figure 4 upper panel). Statisticians typically describe this level of evidence as “barely worth mentioning.” The evidence levels proved robust to variations in the width of the priors. However, we note that ultrawide priors resulted in a moderate evidence level in favor of the null hypothesis (BF10 = 0.2789, BF01 = 3.586) (Figure 4, middle). The evolution of evidence can be seen in Figure 4 (lower) as the sample of observers increases from 1 to 9 (Table 2).

**Table 1:** Performance measures for both report modes during scanning sessions.

Subject number	Saccade				Key-press			
	Hit	False alarm	d’	c	Hit	False alarm	d’	c
1	0.991	0.009	4.720	0.002	0.991	0.009	4.740	0.000
2	0.933	0.009	3.860	0.434	0.938	0.009	3.910	0.415
3	0.990	0.018	4.440	−0.119	0.939	0.077	2.970	−0.060
4	0.846	0.011	3.310	0.635	0.938	0.009	3.900	0.413
5	0.944	0.070	3.060	−0.058	0.958	0.009	4.100	0.317
6	0.891	0.018	3.340	0.437	0.867	0.018	3.220	0.499
7	0.917	0.270	2.000	−0.387	0.878	0.160	2.160	−0.086
8	0.762	0.129	1.840	0.209	0.899	0.025	3.230	0.340
9	0.861	0.094	2.400	0.116	0.911	0.039	3.110	0.212

Hit indicates the hit rate, False indicates the false alarm rate, c indicates an estimate of the decision criteria, and d’ is the estimate of psychophysical performance.

**Table 2:** Summary statistics for both report modes during scanning sessions.

Descriptive statistics	Saccade d’	Saccade c	Keypress d’	Keypress c
Valid	9.000	9.000	9.000	9.000
Missing	0.000	0.000	0.000	0.000
Mean	3.219	0.141	3.482	0.228
Std. error of mean	0.339	0.107	0.253	0.074
Std. deviation	1.016	0.322	0.759	0.223
Variance	1.033	0.104	0.575	0.050
Minimum	1.840	−0.387	2.160	−0.086
Maximum	4.720	0.635	4.740	0.499

**Figure 5:**
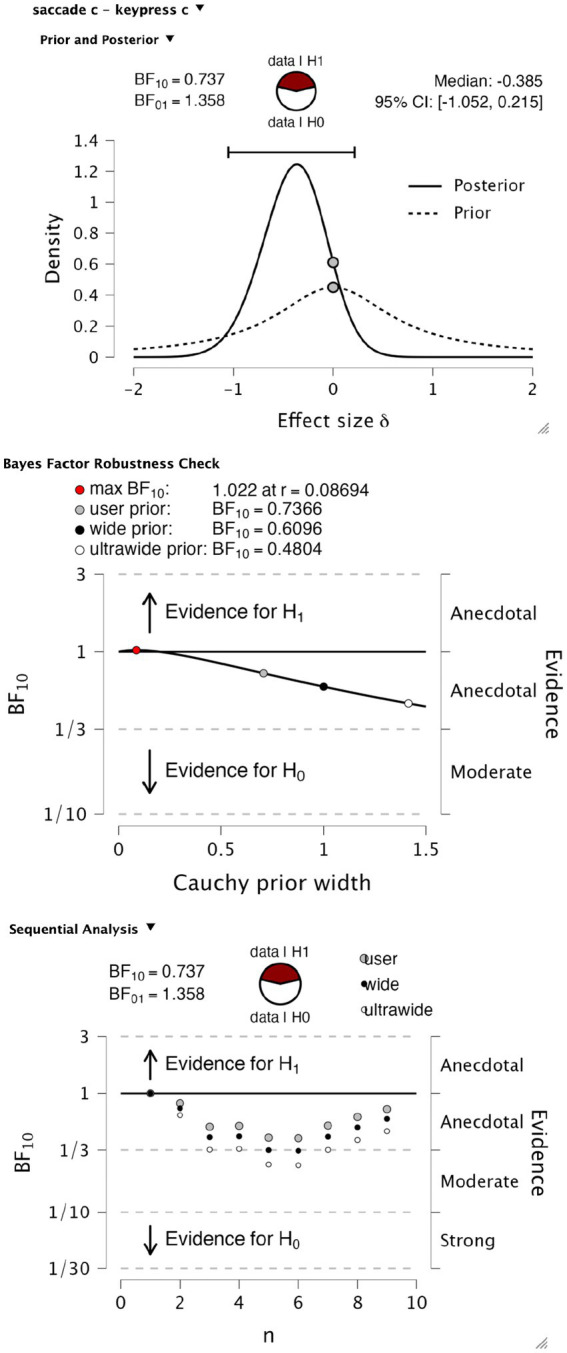
Bayesian *t*-test on criterion values. Upper panel, *t*-test information on prior and posterior values. The middle panel shows a Bayes factor robustness test for the same test. Lower panel, sequential analysis of evidence levels over observer recruitment.

We explored bias as another possible difference in psychophysical performance that report modality could influence. To test for differences in perceptual bias, we performed a Bayesian paired *t*-test testing the null hypothesis of no difference in criterion value against an alternate hypothesis of a difference of either sign, again with default Cauchy priors with a scale parameter of 0.707. We found anecdotal evidence in favor of the null hypothesis (BF10 = 0.737, BF01 = 1.358), with a median effect size of −0.385 and Bayesian credibility intervals (BCI) of [−1.052, 0.215] (Figure 5, upper). This level of evidence proved to be robust to variations in the width of the priors (Figure 5, middle). Again, the evolution of evidence as observers increase from 1 to 9 is shown in Figure 5, lower.

**Figure 6:**
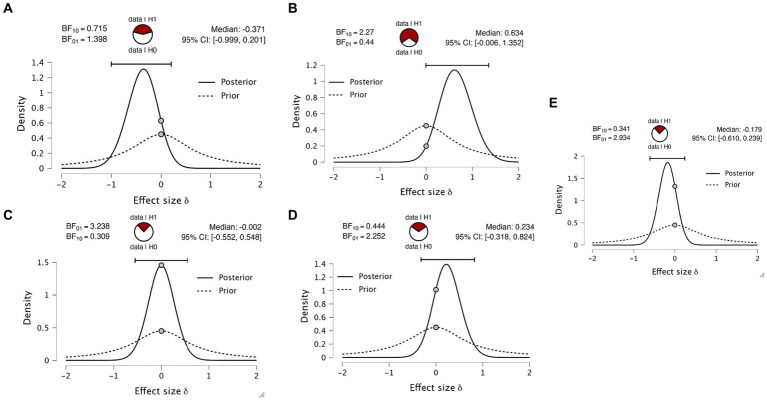
Bayesian paired *t*-tests on Overgaard and Sørensen (2004). Plots indicate the Bayes factors for **(A)** experiment 3 pre-cue, **(B)** experiment 3 post-cue, **(C)** experiment 4 pre-cue, **(D)** experiment 4 post and **(E)** data aggregated data averaging all four experiments and combining with the data obtained from the new dataset reported here.

As mentioned in the introduction, there has (to our knowledge) been only one previous replication attempt of the Marcel (1993) paper (Overgaard and Sørensen, 2004). This study did not report the group statistics relevant to our question. We have taken the opportunity to perform a simple reanalysis of this data. Table 3 displays the d-prime values calculated by Overgaard and Sørensen, 2004, who found a d’ value of 0.91 for eye blinks, 0.85 for button press and 0.18 for verbal response. The only difference between experiments 3 and 4 is the wait time between the stimulus and the report to account for a memory decay effect. Within each experiment, there is a comparison between a pre-cue, where they cued which report modality to use ahead of the stimulus, and a post-cue, where they cued the report modality after the stimulus. We performed the equivalent Bayesian paired two-sided *t*-tests on all four paradigm variants. We obtained anecdotal evidence for all variants (Figures 6A–C), except for experiment 4 in the pre-cue condition, which revealed moderate evidence (Figure 6D, BF01 = 3.2) in favor of the null hypothesis of no difference in d-prime. Both datasets testing for differences in d-prime between eye and hand report conditions are effectively anecdotal or close to anecdotal.

**Table 3:** d-prime values reported in Overgaard and Sørensen (2004).

Participant#	hand_pre-cue_exp3	eye_pre-cue_exp3	delta-pre-cue_exp3	hand_post-cue_exp3	eye_post-cue_exp3	delta_post-cue_exp3	hand_pre-cue_exp4	eye_pre-cue_exp4	delta-pre-cue_exp4	hand_post-cue_exp4	eye_post-cue_exp4	delta_post-cue_exp4
1	2.850	2.600	−0.250	3.200	2.300	−0.900	0.600	2.350	1.75	2.6	2.7	0.1
2	1.900	0.000	−1.900	1.700	0.100	−1.600	2.600	0.000	−2.6	2.85	2.25	−0.6
3	0.900	2.600	1.700	3.200	2.800	−0.400	3.200	1.200	−2	2.15	2.55	0.4
4	1.650	2.450	0.800	2.450	2.200	−0.250	−0.600	2.500	3.1	1.5	1.75	0.25
5	0.150	−1.200	−1.350	0.700	0.300	−0.400	1.950	3.350	1.4	2.2	2	−0.2
6	3.000	3.000	0.000	2.450	2.800	0.350	0.500	2.650	2.15	3.2	2.65	−0.55
7	0.700	2.500	1.800	2.400	2.450	0.050	1.750	0.650	−1.1	2.2	2.35	0.15
8	−0.200	1.850	2.050	2.100	2.200	0.100	1.900	−0.100	−2	2.35	2.35	0
9	0.650	2.050	1.400	2.550	1.700	−0.850	1.800	2.400	0.6	2.35	2	−0.35
10	−1.950	1.400	3.350	2.250	1.700	−0.550	2.400	1.150	−1.25	2.4	2.2	−0.2

Experiments 3 and 4 resulted in d-prime values in pre- and post-cue report conditions. Eye blinks (eye) and button presses (hand).

Finally, to integrate the two datasets, we averaged the d-primes across the four paradigm variants, creating a subject-specific d-prime for hand and eye reports. We appended the data obtained in the experimental data first reported here to this averaged data. This analysis gave a group of 19 subjects. Again calculating the same *t*-test, we find only anecdotal evidence for the alternate hypothesis (Figure 6E, BF01 = 2.934).

### Bayes factor design analysis for future studies

**Figure 7:**
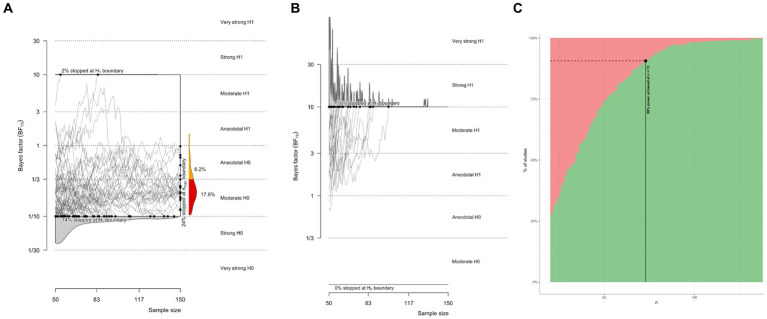
Bayes factor design analysis simulations for future studies. **(A)** Evidence trajectories for experiments where H0 is true. **(B)** Evidence trajectories for experiments where H1 is true. **(C)** Estimated sample size for a power of 90% for correctly detecting a medium effect size.

In light of the inconclusive findings above, it is important to assess how much data would be required to adequately test for the effect of interest. We performed a Bayes factor design analysis (Stefan et al., 2019), as implemented by the Bayes factor design analysis R package (Schönbrodt and Stefan, 2019; R Core Team, 2021). We ran two simulations for two-sided Bayesian paired *t*-tests, one for data generated under the null model (H0 that there is no report modality effect) and data generated under the alternate model (H1 that there is an effect of report modality on psychophysical sensitivity). For the H0 simulation, the effect size was set to 0, with default Cauchy priors on the effect size (sqrt(2)/2), with a minimal number of subjects set at 20 and a maximal number of subjects set at 150. An equivalent simulation was run for H1, where the effect size was set to medium (Cohen’s d = 0.5). For the H0 simulation, the average stopping point was 78, defined as the average number of subjects sampled before hitting a strong evidence threshold of BF = 10 for or against H0. In other words, assuming no effect exists, a strong evidence threshold is reached on average after 78 subjects. 76% of simulated studies correctly hit the strong evidence bound for H0. Only 2% incorrectly hit the strong evidence boundary for H1 (Figure 7A). For the H1 simulation, the average stopping point was 36, meaning that on average, if the true effect size was 0.5, then an average of 36 subjects would be sampled before hitting a strong evidence threshold. 100% of simulated studies correctly hit the strong evidence bound for H1 (Figure 7B). The estimated sample size for 90% of correct detection of H1 was 73 (Figure 7C). This provides some perspective on why the evidence is inconclusive for the small samples presented in the experiments above. Based on simulating different designs, we recommend an optional stopping design, with a minimum of 20 subjects and a maximum of 150 subjects, stopping data acquisition whenever a strong evidence threshold is reached. This policy provides a very large chance of correctly detecting a medium-sized effect if it exists (>99%) and a defensible chance of correctly detecting the absence of an effect (>75%).

## Discussion

### Summary of results

We present the results of two studies that asked if report modality impacts psychophysical sensitivity. Comparing hand and eye reports in similar ways, the results of both studies failed to show even moderate evidence for or against the hypothesis for such an effect. The results presented here remain largely inconclusive; as such, they offer no update to our credences for or against the existence of these report-dependent phenomena. We discuss several limitations of the experiments analyzed, and we end with recommendations for future experiments.

### Sample size and Bayes factor design analysis

An obvious limitation is the small sample size that was obtained. Unfortunately, this is a dataset that was collected a long time ago. Otherwise, it would have been more pragmatic to increase the sample size, for instance, under an optional stopping design. Nonetheless, we performed a Bayes factor design analysis to estimate how many subjects would need to be sampled to reach a power of 90%, defined as a 90% chance of obtaining strong evidence for H1, given that H1 generated the data with an effect size of 0.5. Sample size estimation suggested 73 subjects would be required for this, and even more if we were to obtain a good chance of correctly inferring null effects. The sample size estimate is far from what was obtained in this study and may provide some perspective on why inconclusive results were observed. To test for report modality effects of this kind, we think it is important to be able to infer the null, and thus, we recommend an optional stopping design with an upper limit of at least 150 subjects. According to our simulations, this sampling policy would yield a 75% chance of correct inference on a true null.

### Ceiling effect

Another factor that may have contributed to inconclusive findings is that the hit rate of the main experiment was quite high for both the hand and eye report conditions (approximately 90%). While this leaves some room for performance improvements above 90% and more room for performance decreases downwards, we may have achieved greater sensitivity to any report effect if the task was harder. Going forward, we recommend staircasing psychophysical performance to a lower level of 75% for one of the report modalities before performing the experiment.

### Modeling of report-semantic mappings

Another minor limitation is that the mapping between present versus absent reports and the laterality of the action was counterbalanced across subjects. However, this was not modeled in the statistical analysis. Due to the provenance of the data, we do not have access to the counterbalancing information, so unfortunately, this could not be modeled. This should be included in any future testing.

### Why this question remains important

Report modality is still not typically considered an important factor in the experimental design of perceptual studies. The potential demonstration of RDPP would elevate report modality as an important factor in designing perceptual experiments. This would challenge our current models of perception and provoke new research into the mechanisms underlying these effects. For this reason, we advocate for this experimental question to be empirically resolved.

### Conclusion

The data presented here show no substantive evidence of whether report modalities influence sensory perception. Nevertheless, our attempt to answer this question exposes an overlooked question that remains necessary to answer.

## Data availability statement

The raw data supporting the conclusions of this article will be made available by the authors, without undue reservation.

## Ethics statement

The studies involving humans were approved by the National Hospital for Neurology and Neurosurgery Ethics Committee, London, UK. The studies were conducted in accordance with the local legislation and institutional requirements. The participants provided their written informed consent to participate in this study.

## Author contributions

MO conceived of the idea and wrote the manuscript. OH conceived of the idea, ran the experiment, analyzed data, and wrote the manuscript. BR conceived of the idea, ran the experiment, and analyzed data. All authors contributed to the article and approved the submitted version.

## Footnotes

^1^The original citation year is 1993, the online version of the same book chapter is dated 2007.

^2^Note that Marcel (1993) did not explicitly compute d’, however these were retrospectively calculated and reported in Overgaard and Sørensen (2004). Because the data cannot be fully reconstructed from the original paper, the statistical tests on the differences in d’ cannot be computed.

^3^www.vislab.ucl.ac.uk

^4^www.mathworks.com
